# Genetic Analysis and Molecular Mapping of the Quantitative Trait Loci Governing Low Phytic Acid Content in a Novel LPA Rice Mutant, PLM11

**DOI:** 10.3390/plants9121728

**Published:** 2020-12-08

**Authors:** Prem Chand Gyani, Haritha Bollinedi, Subbaiyan Gopala Krishnan, Kunnummal Kurungara Vinod, Archana Sachdeva, Prolay Kumar Bhowmick, Ranjith Kumar Ellur, Mariappan Nagarajan, Ashok Kumar Singh

**Affiliations:** 1 Division of Genetics, ICAR-Indian Agricultural Research Institute, New Delhi 110012, India; pcg1989@gmail.com (P.C.G.); haritha.agrico@gmail.com (H.B.); krish.icar@gmail.com (S.G.K.); kkvinodh@gmail.com (K.K.V.); prolaybhowmick@gmail.com (P.K.B.); ranjithellur@gmail.com (R.K.E.); 2 Division of Biochemistry, ICAR-Indian Agricultural Research Institute, New Delhi 110012, India; archanas@iari.res.in; 3 Rice Genetics and Breeding Research Centre, ICAR-Indian Agricultural Research Institute, Aduthurai 612101, India; head_aduth@iari.res.in

**Keywords:** rice, low phytic acid, genetic analysis, QTL mapping, myo-inositol, phosphatidylinositol

## Abstract

Breeding rice varieties with a low phytic acid (LPA) content is an effective strategy to overcome micronutrient deficiency in a population which consume rice as a staple food. An LPA mutant, Pusa LPA Mutant 11 (PLM11), was identified from an ethyl methane sulfonate (EMS)-induced population of Nagina 22. The present study was carried out to map the loci governing the LPA trait in PLM11 using an F_2:3_ population derived from a cross between a high phytic acid rice variety, Pusa Basmati 6, with PLM11. The genotyping of the F_2_ population with 78 polymorphic SSR markers followed by the estimation of phytic acid content in the seeds harvested from 176 F_2_ plants helped in mapping a major QTL, *qLPA8.1*, explaining a 22.2% phenotypic variation on Chromosome 8. The QTL was delimited to a 1.96 cM region flanked by the markers RM25 and RM22832. Since there are no previous reports of a QTL/gene governing the LPA content in rice in this region, the QTL *qLPA8.1* is a novel QTL. *In silico* analysis based on the annotated physical map of rice suggested the possible involvement of a locus, *Os08g0274775*, encoding for a protein similar to a phosphatidylinositol 3- and 4-kinase family member. This needs further validation and fine mapping. Since this QTL is currently specific to PLM11, the linked markers can be utilized for the development of rice varieties with reduced phytic acid (PA) content using PLM11 as the donor, thus enhancing the bioavailability of mineral micronutrients in humans.

## 1. Introduction

Micronutrient malnutrition is one of the most serious challenges to humanity, as two-thirds of the world’s population is at risk of deficiency in one or more essential mineral elements [1]. It is more rampant among populations in developing countries who are dependent on a rice-based diet for their caloric requirements. Mostly, rice is consumed as milled grains or white rice which is either cooked directly or made into flour or batter to make various preparations. White rice is primarily composed of starchy endosperm and is obtained by the removal of the aleurone layer after polishing the brown rice. About 90% of the iron (Fe) and zinc (Zn) content in rice grain is localized in the aleurone layer. Mineral elements such as Fe and Zn are essential micronutrients in human diet, deficiency of which causes micronutrient malnutrition, infamously known as hidden hunger. The removal of aleurone makes the rice grains deficient in most of these essential micronutrients, predisposing rice eaters to hidden hunger [2,3]. Fe is essential for resistance against infection, enhanced work capacity, cognitive development, and productivity [4], while Zn enhances cellular growth and differentiation, and their deficiency leads to immune dysfunction, impaired growth, increased morbidity and mortality, unfavourable pregnancy outcomes, and deviant neurobehavioral development [5].

The consumption of brown rice can help combat nutritional deficiencies among the rice eating population, as it is nutritionally superior to white rice. Besides being rich in Fe and Zn, it is also rich in other mineral nutrients, such as phosphorus (P), potassium (K), manganese (Mn), magnesium (Mg), calcium (Ca), etc. Although encouraging brown rice consumption can alleviate micronutrient malnutrition to a large extent, the presence of phytic acid (PA) as an anti-nutritional factor remains a major hindrance in the popularization process among the major rice eating population in Asia. The PA, also known as myo-inositol 1,2,3,4,5,6-hexakisphosphate (C_6_H_18_O_24_P_6_; IP6) or phytate as a salt, is a major phosphorus storage compound in most of the seeds including cereal grains. PA takes up to 7% of the kernel dry weight and accounts for more than 70% of the total grain phosphorus. Owing to the presence of high density of negatively charged phosphate groups, PA is a strong chelating agent for multivalent metal ions, especially Zn and Fe. It forms insoluble salts with these cations that remain unabsorbed in the gastrointestinal tract, leading to the poor mineral bioavailability in the body [6]. Therefore, lowering the PA content in brown rice is one of the low-cost and sustainable approaches to increase the bioavailability of essential micronutrients in rice. Use of low PA (LPA) grains can thereby aid in ameliorating Fe and Zn malnutrition among rice eating populations in poor and developing countries.

To tackle the challenge of PA accumulation in rice grains, it is imperative to understand the innate variability for grain PA content in rice germplasm and the identification of LPA genotypes/mutants. Besides, understanding the basis of LPA trait, including the genes and the governing pathways, can pave way for the development of LPA rice varieties. Over the past decade, several LPA mutants have been identified from induced mutagenesis in crops such as maize, barley, wheat, soybean, etc. [7]. Compared to maize and barley, limited progress has been made in rice towards identifying genes and delineating the pathways affecting PA biosynthesis. Furthermore, the use of induced mutagenesis for developing LPA mutants in rice has not found significant achievements, except for a few recent studies [8].

We have used induced mutagenesis to induce variation in grain PA content in a landrace derived upland cultivar, Nagina 22 (N22) in the present study. We report here the identification of a novel LPA mutant, having a 70% reduction in grain PA content. Further, we have mapped the QTL governing the LPA trait in PLM11 using an F_2:3_ population derived from the cross between PLM11 and a high PA Basmati cultivar, Pusa Basmati 6 (PB6).

## 2. Results

### 2.1. Identification of the Novel LPA Mutant

**Figure 1:**
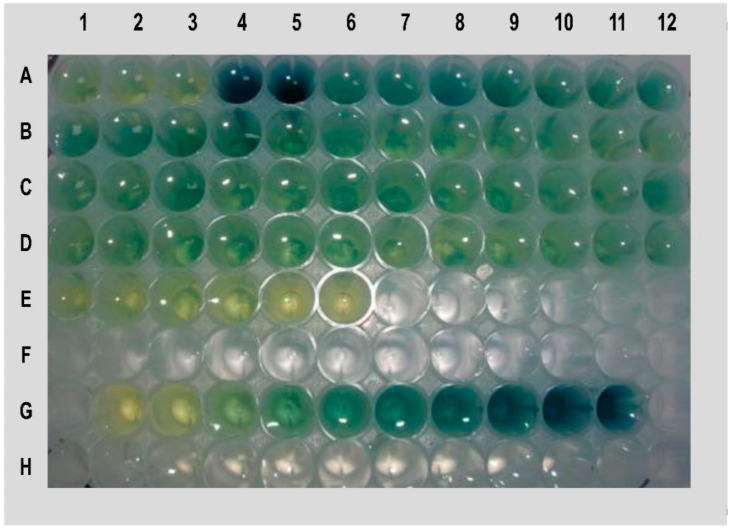
Picture showing the results of high inorganic phosphorus (HIP) assay used for screening the ethyl methane sulfonate (EMS) induced mutants of Nagina 22 (N22) for low phytic acid (LPA) content. Darker the intensity of blue colour, lesser is the phytic acid concentration. Wells 1A, 2A and 3A represent the wild type, N22 while 4A to E6 represent N22 mutants; 4A and 5A are LPA mutants, of which 5A is the mutant used in this study, PLM11; G1 to G11 represent standards.

**Figure 2:**
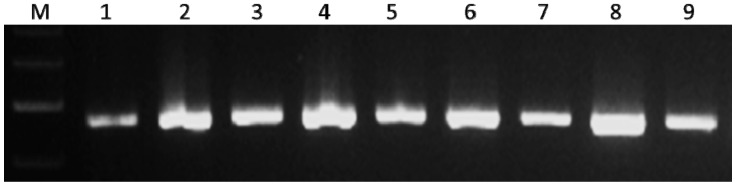
Representative gel picture showing the presence of wild type allele of known low phytic acid (LPA) genes in the mutant, PLM11. The picture shows cleaved amplified polymorphic sequence (CAPS) assay with the marker, LPA1_CAPS specific to *lpa1-1* allele. The mutant PLM11 and the wild type, N22 shows similar banding pattern with a monomorphic amplicon of fragment size 184bp. M, DNA ladder; 1, wildtype N22; 2–9, LPA mutants.

PLM11 was identified based on the screening of 91 ethyl methane sulfonate (EMS) induced mutants of the *Aus* cultivar, N22, through a colorimetric assay. The assay was based on the presence of high inorganic phosphorus (HIP) in grains as an indirect indicator of LPA. The presence of HIP was indicated by the occurrence of a dark blue (molybdenum blue) colour in the assay panel. When N22 mutants were screened, one of the mutants expressed an intense blue colour indicating the presence of HIP (Figure 1). This mutant was named as Pusa LPA mutant 11 (PLM11). This mutant had an average grain PA content of 1.2 mg/g as against the PA content of about 4.0 mg/g in N22, recording a 70% reduction. Apart from the LPA grains, no undesirable agronomic features were noticed in PLM11. Subsequent PCR assay of PLM11 using gene-based markers from known LPA mutants such as *lpa1*, *LPA1_InDel*, and *LPA1_CAPS* [9] produced wild-type alleles (Figure 2), indicating that the novelty of the LPA phenotype of PLM11 could be of a different mutation.

### 2.2. Phenotypic Variation in Parents and the F_2:3_ Population

**Figure 3:**
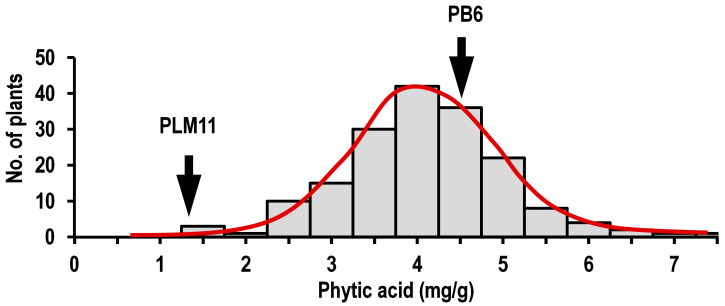
Frequency distribution for the grain concentration of phytic acid (mg/g) in the F_2_ population from the cross, PB6/PLM11.

The molecular mapping of the LPA trait of PLM11 was performed using an F_2_ mapping population from the cross, PLM11/PB6. The parents exhibited a significant contrast for the PA content in brown rice. PB6 recorded a high PA content of 4.6 ± 0.43 mg/g, while it was as low as 1.2 ± 0.16 mg/g in PLM11. The F_2:3_ seeds exhibited significant variation for PA ranging from 1.2 to 7.2 mg/g, with a mean value of 3.83 mg/g. LPA showed a normal distribution in the F_2:3_ population, signifying its quantitative inheritance (Figure 3).

### 2.3. Development of Linkage Map Using Simple Sequence Repeat (SSR) Markers

**Figure 4:**
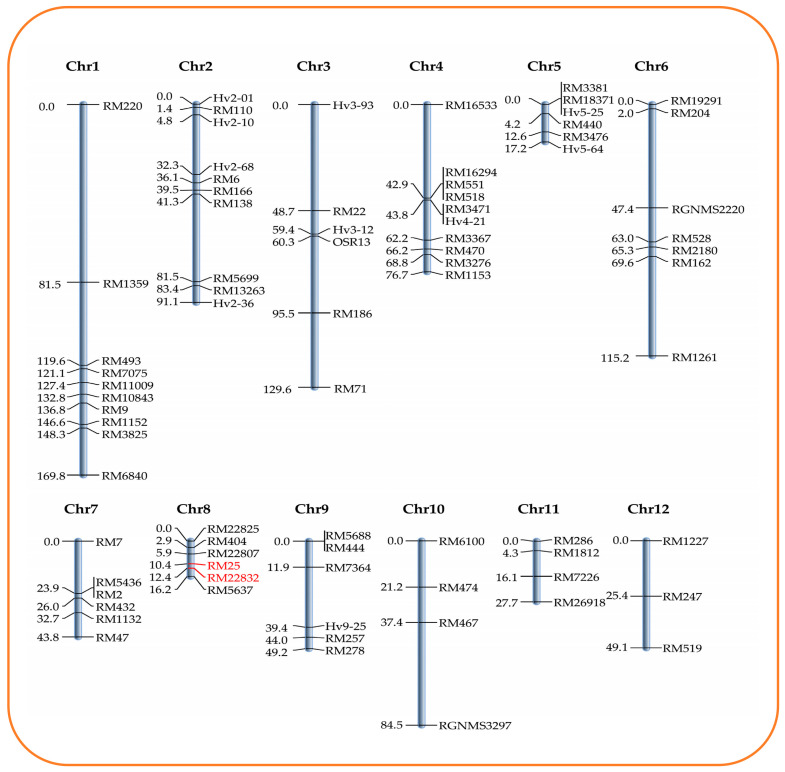
Linkage map based on the F_2_ population from the cross, PB6/PLM11, showing chromosome numbers, distance (cM), and marker names.

A total of 331 SSR markers spanning across all the 12 rice chromosomes were used for genome-wide parental polymorphism survey between the parents, PB6 and PLM11, out of which 78 SSR markers were found to be polymorphic (Table 1). A list of polymorphic markers is provided in Supplementary Table S1. The average polymorphism was 25.1%. The highest number of polymorphic markers was recorded in chromosome 7, with a polymorphism of 46.2%, whereas the lowest polymorphism was observed in chromosome 11 (18.2%). The chromosome-wide marker density ranged from 2.7 cM/marker in chromosome 8 to 21.6 cM/marker in chromosome 3. The genotyping of the F_2_ population was conducted using a total of 78 polymorphic markers. All the markers showed Mendelian segregation as determined by the χ^2^ test for goodness of fit. These were used to develop a molecular linkage map using the software “QTL IciMapping” [10]. The linkage map was constructed with 78 SSR markers spanning 870.21 cM and distributed across 12 chromosomes, with an average distance of 11.4 cM between the markers (Figure 4).

### 2.4. Mapping the QTL for LPA Content and In Silico Analysis

**Figure 5:**
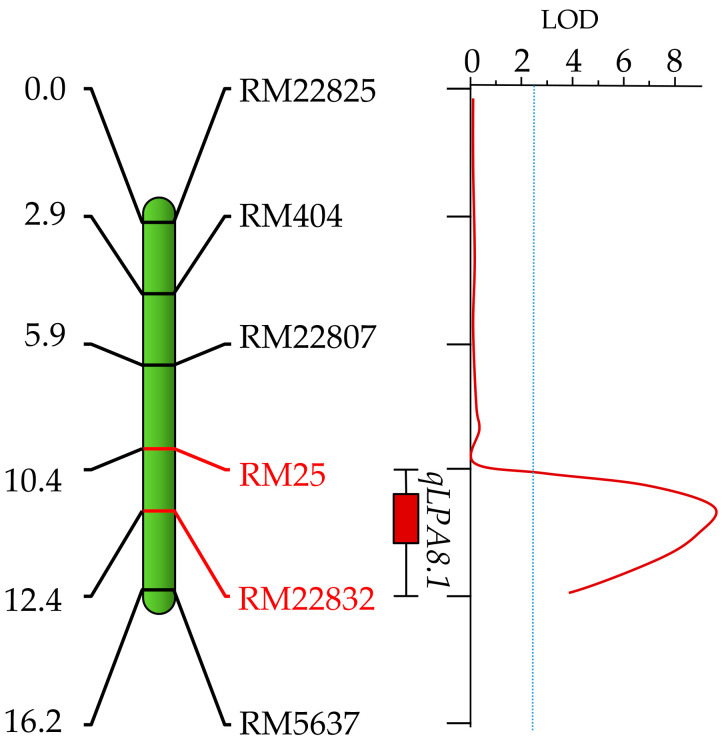
Linkage map of chromosome 8 of rice from PB6/PLM11 population showing the position of a major quantitative trait locus (QTL), *qLPA8.1*, governing low phytic acid (LPA) content identified from the LPA rice mutant, PLM11.

QTL analysis using the inclusive composite interval mapping (ICIM) approach identified a major QTL on chromosome 8, explaining 22.2% of the total phenotypic variation explained (PVE) for grain LPA content. We name this QTL as *qLPA8.1*, which is flanked by the markers RM25 and RM22832 and separated by a distance of 1.96 cM (Figure 5). *qLPA8.1* had a significantly high LOD value of 9.08, with the favourable allele associated with LPA derived from the LPA mutant, PLM11. The QTL recorded an additive effect of 0.662 mg/g and a dominance effect of 0.084 mg/g of phytic acid (Table 2). An *in silico* analysis of the QTL region based on the annotated rice genome suggested that the genomic region of this QTL has an approximate physical distance of 7 Mb. Search for the annotated genes within this region could trace 530 genes (Supplementary Table S2). A refined search on annotated functions of the genes, revealed the presence of a gene, *Os08g0274775*, that encoded for a protein similar to phosphatidylinositol 4-kinase (PtdIns4K) family member, with the possible connotations of involvement in the lipid-dependent pathway of PA biosynthesis. However, no functional characterisation of this gene is available.

## 3. Discussion

Nowadays, the consumption of whole-grain foods is gaining popularity owing to their health benefits. The polished white rice, which is popular among rice-based diets, is devoid of precious nutrients, including essential minerals and vitamins which are lost in the process of milling. Hence, white rice consumption is predisposing the rice eating population to micronutrient malnutrition. The health-conscious public is changing their preferences to brown rice consumption, given its richness in minerals, vitamins, dietary fibre, and other antioxidant compounds with beneficial health effects. Nevertheless, the presence of anti-nutritional factor such as PA remains a major challenge in popularizing the consumption of brown rice among the rice eating population. The screening of rice germplasm for the variability of PA and the identification of LPA genotypes in diverse genetic backgrounds could help in the molecular mapping of the gene(s) governing LPA. It enables the elucidation of the PA metabolic pathway as well as aids in rice biofortification for reducing PA, thereby enhancing the micronutrient bioavailability. In the present study, we have identified an EMS-induced LPA mutant, PLM11, in the background of an upland *Aus* rice variety, Nagina 22. The mutant showed about a 70% reduction in PA phosphorous, while the first reported LPA mutant in rice, “Kaybonnet” lpa1-1, which was a nonlethal single recessive mutant, had an approximate 45% reduction in seed PA and a molar equivalent increase in inorganic phosphorus as compared to the wild type [11]. Another LPA mutant of an *indica* type rice, developed by the mutation of 2-phosphoglycerate kinase (2-PGK) gene (*Os-lpa-XQZ-1*), showed a 12–35% reduction in PA compared to the wild type [12]. A mutant of myo-inositol kinase (MIK) gene, *Os-lpa-XS110–1*, showed a 46% reduction in the PA content [13]. Whereas, a mutation in *Os-lpa-XS110–2*, which was found similar to the *lpa1*-type mutation in maize, showed a reduction of 23% in the PA content along with the increase in inorganic phosphorus [13,14,15]. Among the eight LPA mutants in rice isolated by Liu et al. [15], one mutant that had mapped on chromosome 3 was found to be orthologous to the MIK gene in the maize *lpa3* mutant, whereas another one was allelic to the rice *lpa1* mutant. In a recent GWAS study of the world rice core collection (WRC), inositol-3-phosphate synthase (*INO1*) was found to be closely localized to a significant SNP, suggesting that the gene itself, as well as its regulation, was important for determining the PA content in rice [16]. Our investigation revealed that the causative locus of LPA trait in PLM11 lies on chromosome 8, which was different from the previously reported genes for PA accumulation in rice grains. We also found that markers related to the *lpa1-1* allele of the Kaybonnet *lpa1-1* mutant and its allelic variant *Os-lpa-XQZ-1* (*lpa1-2*), both the CAPS markers (*LPA1_CAPS*), as well as the InDel marker (*LPA1_InDel*) specific to the *lpa1-2* allele [9], were found to be monomorphic between PLM11 and its wild-type genotype, N22. As these are functional markers based on the polymorphism providing the functionality of LPA in the earlier reported mutants, PLM11 was confirmed to carry the wild *LPA1* allele. These observations provide us with convincing evidence that PLM11 harbours a novel mutation, which is different from earlier reported mutations in rice.

Molecular mapping for the identification of genomic regions determining LPA is an important step towards its use in rice improvement aimed at the development of LPA rice varieties. In the present study, QTL mapping was accomplished using a robust QTL mapping approach, ICIM, an improvement over the frequently used composite interval mapping (CIM) algorithm. It attempts a two-step mapping protocol, wherein significant markers are identified in the first step through step-wise regression, followed by the adjustment of phenotypic values using marker variables retained in the regression model [17]. A major QTL, *qLPA8.1*, explaining 22.2% of the phenotypic variation for the grain PA content, was identified in this study derived from PLM11. Earlier, through QTL mapping approach, Stangoulis et al. [18] reported two QTLs, one in chromosome 5, flanked by markers RM305 and RM178 and another in chromosome 12, flanked by markers RM247 and RM179, explaining 24.3% and 15.4% of the PV, respectively.

*In silico* analysis of the QTL region, *qLPA8.1*, revealed the presence of 530 genes in the annotated rice genome, within the 7Mb region spanning the QTL flanking markers. These genes have no annotated functions related to PA biosynthesis or accumulation. However, one gene on chromosome 8, *Os08g0274775*, was annotated to encode for a protein similar to a PtdIns4K family member. PtdIns4K is known to be involved in the lipid-dependent pathway of PA biosynthesis. However, this assumption requires further validation in the novel LPA mutant, PLM11. In a recent genome wide association study [19], among the 12 marker-trait associations identified on nine different chromosomes, one locus on chromosome 8 was found associated with LPA content. When compared, we could find that this locus neither co-located with the *qLPA8.1* nor with any known PA biosynthesis or accumulation-related genes. These results indicate that LPA can also be under the genetic control by mutation within unknown genes or *cis*-regulatory regions that may affect the expression of PA biosynthesis genes. New molecular mechanisms regulating the grain PA content could also be present in this region. Further insight is required for better understanding of the genetic architecture and underlying mechanism of PA biosynthesis and accumulation in rice grain in PLM11. Having identified the QTL, *qLPA8.1*, associated with the LPA trait in PLM11, the associated markers can now be used for the selection of the trait, when PLM11 is used as the donor of LPA trait to other elite backgrounds. This opens up the path to introgress the LPA trait in rice varieties, ensuring better bioavailability of the micronutrients. Therefore, this can augment the biofortification programme currently ongoing in rice worldwide, without increasing the micronutrient content in the rice grains. The fact that PLM11, the novel LPA mutant has no undesirable effect on crop fitness is an additional bonus, as it can help in the fight against hidden hunger.

## 4. Materials and Methods

### 4.1. Plant Materials

The plant materials used for screening the LPA included a set of 91 advanced-generation EMS mutants in the background of Nagina 22. PLM11 is an LPA mutant identified in the present study. Pusa Basmati 6 (PB6) is a Basmati rice variety with an extra-long slender grain developed at the ICAR-IARI, New Delhi. It has an intermediate amylose content, a low gelatinization temperature (or high alkali spreading value), and a very long kernel length after cooking [20]. For genetic analysis and molecular mapping, an F_2_ population was developed through a cross between the LPA mutant, PLM11, and a Basmati rice variety, Pusa Basmati 6 (PB6). The mapping population consisted of 176 F_2_ plants which were grown at ICAR-IARI, New Delhi, during *Kharif* 2019 following standard agronomic practices. The seeds harvested from each of the 176 individual F_2_ plants were used for phenotyping the PA content.

### 4.2. HIP Assay for Screening of EMS Mutants

The systematic screening of 91 advanced-generation EMS mutants (in the background of Nagina 22) for their phytic acid content was carried out at the Division of Genetics, ICAR-IARI, New Delhi, using the HIP assay. For sample preparation, ground brown rice kernels weighing 100 mg from each genotype were weighed and placed in 2 mL Eppendorf tubes; subsequently, 2 mL of 0.65 *M* HCl was added to the same. The tubes were continuously shaken for 10–12 h at 120 rpm at room temperature and then were centrifuged for 5 min at 12,000 rpm. Then, 500 µL of the extract of each genotype were transferred to a fresh 2 mL Eppendorf tube for PA estimation, and to a 15 mL tube for inorganic P estimation. For quantification, equal volumes of quantitative standards were used for both phytate as well as Pi. Phytic acid dodecasodium salt from rice (Sigma) was used as phytate standard and KH_2_PO_4_ (HiMedia) was used as a Pi standard. The freshly prepared Pi reagent was used, which consisted of 1 part 0.02 M ammonium molybdate, 1 part 0.57 M ascorbic acid, 1 part 3 M sulfuric acid, and 2 parts distilled H_2_O. For Pi estimation, 1 mL of distilled H_2_O and 1 mL of Pi reagent were added to each tube. After 15 to 20 min, a blue colour developed at room temperature, and then its optical density was measured at 820 nm (OD 820) using a 96-well plate.

### 4.3. Phenotyping of Mapping Population

The seeds were dehusked by a palm husker and brown rice was obtained. These brown rice samples from parental lines and 176 F_2:3_ seeds were phenotyped using the protocol described by Megazyme’s K-PHYT kit. For sample extraction, 1 g of sample material was weighed into a 75 mL glass beaker and 20 mL of 0.66 M hydrochloric acid was added to it. The beaker was covered with foil and vigorously stirred at room temperature for at least 3 h, preferably overnight. A total of 1 mL of the extract was transferred to a microfuge tube of 1.5 mL and centrifuged for 10 min at 13,000 rpm. Instantly, 0.5 mL of the supernatant was transferred to a fresh microfuge tube of 1.5 mL and a further 0.5 mL of 0.75 M sodium hydroxide solution was added to neutralize the samples. The enzymatic dephosphorylation reaction for both free and total phosphorous was performed in a neutralised sample extract. Free phosphorous was quantified using following steps: (1) 0.50 μL of sample extract, 0.62 mL distilled water (DW), and 0.2 mL buffer were mixed and incubated for 10 min in a water bath set at 40 °C. (2) Then, 0.02 mL of buffer and DW were added, vortexed, and re-incubated for 15 min in a water bath set at 40 °C. (3) Finally, to terminate the reaction, 0.3 mL of trichloroacetic acid (50%) was added. Similarly, for the quantification of total phosphorous in addition to the procedure described for free phosphorous, 0.02 mL of each phytase and alkaline phosphatase (ALP) were added in step 1 and step 2, respectively. Terminated reactions of both free phosphorous and total phosphorous were centrifuged for 10 min at 13,000 rpm. For the colorimetric determination of phosphorous, 1 mL of the supernatant was carefully pipetted out in a microfuge tube, then 0.5 mL of colouring reagent was added, vortexed, and incubated for 1 h in a water bath set at 40 °C. After an hour, the same was vortexed, 1 mL was transferred to a semi-micro cuvette, and the absorbance was taken at 655 nm (A655). Finally, the concentration of the phosphorous and phytic acid in the samples were calculated using the absorbance (A655) values for free phosphorus and total phosphorus.

### 4.4. Construction of Molecular Linkage Map

For the parental polymorphism survey between the contrasting parental genotypes namely, PB6 (high PA content) and PLM11 (low PA content), a total of 331 simple sequence repeat (SSR) markers providing genome wide coverage were used. The total genomic DNA from 176 individual F_2_ plants was extracted using the Murray and Thompson [21] protocol. Thermal cycler (Applied Biosystems^®^ Veriti^®^, Foster City, CA, USA) was used to perform polymerase chain reaction (PCR). The reaction mixture with a total volume of 10 μL consisted of template DNA (30 ng), 5 pmol of each primer (synthesized by Sigma Inc., USA), MgCl_2_ (1.5 mM), 0.2 mM of dNTPs (MBI, Fermentas, Lithuania), and 0.5 U of Taq polymerase (Bangalore Genei, Bangalore, India). PCR comprised of first cycle of denaturation for 5 min at 95 °C, followed by 35 cycles having different stages such as denaturation for 30 s at 95 °C, annealing for 30 s at 55 °C and extension for 1 min at 72 °C. The final extension was for 7 min at 72 °C. Metaphor™ Agarose gel (3.5% to 4.0%) containing 0.1 mg/mL of ethidium bromide (Amresco, Albany, NY, USA) was used to resolve amplicons. The amplified products were electrophoresed for 2 h along with a 50 bp ladder (MBI, Fermentas, Vilnius, Lithuania) and parents. Finally, an ultraviolet transilluminator (Gel DocTM XR + Imager, Bio-Rad Laboratories Inc., Hercules, CA, USA) was used to visualize the amplified bands. A total of 78 well-distributed polymorphic markers were used for genotyping 176 F_2_ plants along with the parental lines. Marker allele segregation data scored from the gels was used for the Chi-square (χ^2^) analysis for testing the goodness of fit to the expected segregation ratio. Those markers which did not fit well to the ratio were treated as distorted markers. The markers showing segregation distortion were removed from further analysis. QTL IciMapping software [10] was used to construct a linkage map. Kosambi’s mapping function [22] was used to calculate the map distances.

### 4.5. QTL Mapping

The inclusive composite interval mapping (ICIM) function in the QTL IciMapping v4.1 (www.isbreeding.net) was used to perform the QTL mapping. It was used to determine the precise location of putative QTL and the estimation of QTL effects such as PVE, log-likelihood ratio (LOD) score, additive effect, and dominance effect of the QTL locus [23]. Permutation test involving 3000 runs at *p* = 0.05 level of significance was used to determine the threshold LOD value. The test statistic employed for LOD was −2ln (L0/L1), where L0/L1 is the ratio of the likelihood under the null hypothesis i.e., absence of QTL in the interval and the alternative hypothesis i.e., presence of QTL in the interval. At positions where the LOD score exceeded the corresponding significant threshold, QTLs were found to exist. At significant LOD, the peak genetic effects, position, and phenotypic variation percentage of the QTLs were estimated. Nomenclature of QTL was performed as described in McCouch et al. [24].

## 5. Conclusions

We identified a novel LPA mutant PLM11 in rice, following an EMS induced mutagenesis on the cultivar, Nagina 22. Genetic analysis and molecular mapping of LPA trait in PLM11 identified a major QTL, *qLPA8.1*, explaining 22.2% of the PVE on chromosome 8. The PCR based markers, RM25 and RM22832, were identified flanking the QTL. These markers can be used in the marker-aided introgression aimed at the development of LPA rice varieties, using PLM11 as the donor of the trait. The *in silico* analysis was suggestive of a locus, *Os08g0274775*, encoding for a protein similar to phosphatidylinositol 4-kinase family member, with a possible activity in the lipid dependent biosynthesis of PA. The QTL region requires further fine mapping and identification of the candidate gene responsible for driving LPA in the mutant, PLM11. Subsequent cloning and functional validation of the candidate gene within this QTL region would facilitate the understanding of the regulation of PA biosynthesis and accumulation in rice. It might also help to address the questions on the potential role of inositol lipid-dependent biosynthetic pathways in determining the phytic acid content of seeds and other plant tissues.

## Figures and Tables

**Table 1:** Details of the markers used for the construction of a linkage map based on the F_2_ population of the cross, PB6/PLM11.

Chromosome	Total No. of Markers	No. of Polymorphic Markers Used for Genotyping F_2_s	Percent Polymorphism	Map Length (cM)	Marker Density (cM/marker)
1	39	10	25.6	169.8	17.0
2	42	10	23.8	91.1	9.1
3	24	6	25.0	129.6	21.6
4	51	10	19.6	76.7	7.7
5	31	6	19.4	17.2	2.9
6	26	7	26.9	115.2	16.5
7	13	6	46.2	43.8	7.3
8	29	6	20.7	16.2	2.7
9	28	6	21.4	49.2	8.2
10	15	4	26.7	84.5	21.1
11	22	4	18.2	27.7	6.9
12	11	3	27.3	49.1	16.4
**Total**	331	78	25.1	870.2	11.4

cM—centimorgans.

**Table 2:** Details of the major QTL identified for LPA trait in the F_2_ population derived from the cross, Pusa Basmati 6/PLM11 through inclusive composite interval mapping (ICIM).

Method	QTL	Chr	Position (cM)	Left Marker	Right Marker	LOD	PVE (%)	Additive Effect *	Dominance Effect *
ICIM	*qLPA8.1*	8	12	RM25	RM22832	9.06	22.21	0.7	0.084

ICIM: Inclusive Composite Interval Mapping; QTL: quantitative trait loci; Chr.: chromosome; PVE: percentage of variance explained; * additive and dominance effects have the same unit as the phenotype (mg/g).

## Supplementary Materials

The following are available online at https://www.mdpi.com/2223-7747/9/12/1728/s1. Table S1: List of polymorphic markers, their physical position and chromosome number used for developing the linkage map from PB6/PLM11 population; Table S2: Comprehensive list of annotated genes identified within the *qLPA8.1* QTL region.

## References

[1] White P.J., Broadley M.R. (2009). Biofortification of crops with seven mineral elements often lacking in human diets—Iron, zinc, copper, calcium, magnesium, selenium and iodine. New Phytol..

[2] Bollinedi H., Vinod K.K., Bisht K., Chauhan A., Krishnan S.G., Bhowmick P.K., Nagarajan M., Rao D.S., Ellur R.K., Singh A.K. (2020). Characterising the diversity of grain nutritional and physico-chemical quality in Indian rice landraces by multivariate genetic analyses. Indian J. Genet..

[3] Bollinedi H., Yadav A.K., Vinod K.K., Gopala Krishnan S., Bhowmick P.K., Nagarajan M., Neeraja C.N., Ellur R.K., Singh A.K. (2020). Genome-wide association study reveals novel marker-trait associations (MTAs) governing the localization of Fe and Zn in the rice grain. Front. Genet..

[4] Alwan N.A., Hamamy H. (2015). Prenatal exposures and short and long term developmental outcomes: Maternal iron status in pregnancy and long-term health outcomes in the offspring. J. Pediatr. Genet..

[5] Hojyo S., Fukada T. (2016). Roles of zinc signaling in the immune system. J. Immunol. Res..

[6] Graham R.D., Welch R.M., Bouis H.E. (2001). Addressing micronutrient malnutrition through enhancing the nutritional quality of staple foods: Principles, perspectives and knowledge gaps. Adv. Agron..

[7] Raboy V. (2007). The ABCs of low-phytate crops. Nat. Biotechnol..

[8] Qamar Z.U., Hameed A., Ashraf M., Rizwan M., Akhtar M. (2019). Development and molecular characterization of low phytate Basmati rice through induced mutagenesis, hybridization, backcross, and marker assisted breeding. Front. Plant Sci..

[9] Zhao H.-J., Liu Q.-L., Ren X.-L., Wu D.-X., Shu Q. (2008). Gene identification and allele-specific marker development for two allelic low phytic acid mutations in rice (*Oryza sativa* L.). Mol. Breed..

[10] Meng L., Li H., Zhang L., Wang J. (2015). QTL IciMapping: Integrated software for genetic linkage map construction and quantitative trait locus mapping in biparental populations. Crop. J..

[11] Larson S.R., Rutger J.N., Young K.A., Raboy V. (2000). Isolation and genetic mapping of a non-lethal rice (*Oryza sativa*), low phytic acid 1 mutation. Crop Sci..

[12] Frank T., Habernegg R., Yuan F.-J., Shu Q., Engel K.-H. (2009). Assessment of the contents of phytic acid and divalent cations in low phytic acid (lpa) mutants of rice and soybean. J. Food Compos. Anal..

[13] Frank T., Meuleye B.S., Miller A., Shu Q., Engel K.-H. (2007). Metabolite profiling of two low phytic acid (lpa) rice mutants. J. Agric. Food Chem..

[14] Goodman C.D., Casati P., Walbot V.A. (2004). Multidrug resistance–associated protein involved in anthocyanin transport in *Zea mays*. Plant Cell.

[15] Liu Q.-L., Xu X.-H., Ren X.-L., Fu H.-W., Wu D.-X., Shu Q. (2007). Generation and characterization of low phytic acid germplasm in rice (*Oryza sativa* L.). Theor. Appl. Genet..

[16] Perera I., Fukushima A., Akabane T., Horiguchi G., Seneweera S., Hirotsu N. (2019). Expression regulation of myo-inositol 3-phosphate synthase 1 (INO1) in determination of phytic acid accumulation in rice grain. Sci. Rep..

[17] Li H., Ye G., Wang J. (2007). A modified algorithm for the improvement of composite interval mapping. Genetics.

[18] Stangoulis J.C., Huynh B.-L., Welch R.M., Choi E.-Y., Graham R.D. (2007). Quantitative trait loci for phytate in rice grain and their relationship with grain micronutrient content. Euphytica.

[19] Perera I., Fukushima A., Arai M., Yamada K., Nagasaka S., Seneweera S., Hirotsu N. (2019). Identification of low phytic acid and high Zn bioavailable rice (*Oryza sativa* L.) from 69 accessions of the world rice core collection. J. Cereal Sci..

[20] Singh V.P., Singh A.K., Atwal S.S., Joseph M., Mohapatra T. (2002). Pusa 1121: A rice line with exceptionally high cooked kernel elongation and basmati quality. Int. Rice Res. Notes.

[21] Murray M., Thompson W. (1980). Rapid isolation of high molecular weight plant DNA. Nucleic Acids Res..

[22] Kosambi D.D. (1944). The estimation of map distances from recombination values. Ann. Eugen..

[23] Wang J.-K. (2009). Inclusive Composite Interval Mapping of quantitative trait genes. Acta Agron. Sin..

[24] McCouch S.R., Cho G.Y., Yano M. (1997). Report on QTL nomenclature. Rice Genet Newsl..

