# Supplementary material for: Genetic Analysis and Molecular Mapping of the Quantitative Trait Loci Governing Low Phytic Acid Content in a Novel LPA Rice Mutant, PLM11
Source: Plants (Basel). 2020 Dec 8;9(12):1728. doi: 10.3390/plants9121728 (PMC7762397; doi:10.3390/plants9121728)
Supplement: Supplementary file 1 [file plants-09-01728-s001.pdf]

Supplementary Materials

Genetic Analysis and Molecular Mapping of the Quantitative Trait Loci Governing Low Phytic Acid Content in a Novel LPA Rice Mutant, PLM11

Prem Chand Gyani, Haritha Bollinedi, Subbaiyan Gopala Krishnan, Kunnummal Kurungara Vinod, Archana Sachdeva, Prolay Kumar Bhowmick, Ranjith Kumar Ellur, Mariappan Nagarajan and Ashok Kumar Singh*

* Correspondence: aks_gene@yahoo.com

Supplementary Table S1. List of polymorphic markers, their physical position and chromosome number

S.No.	Marker Name	Chromosome	Position
1	RM220	1	4.42
2	RM1359	1	7.9
3	RM493	1	12.26
4	RM7075	1	15.11
5	RM11009	1	18.01
6	RM10843	1	13.79
7	RM9	1	23.32
8	RM1152	1	30.09
9	RM3825	1	36.4
10	RM6840	1	43.17
11	Hv2-01	2	0.124
12	RM110	2	1.33
13	Hv2-10	2	4.29
14	Hv2-68	2	26.1
15	RM6	2	29.57
16	RM166	2	34.35
17	RM138	2	35.68
18	RM5699	2	8.98
19	RM13263	2	18.09
20	Hv2-36	2	11.67
21	Hv3-93	3	34.55
22	RM22	3	1.52
23	Hv3-12	3	4.92
24	OSR13	3	7.12
25	RM186	3	28.8
26	RM71	3	8.76
27	RM16533	4	8.63
28	RM16294	4	0.92
29	RM551	4	0.17
30	RM518	4	2.03
31	RM3471	4	6.31
32	Hv4-21	4	15.57
33	RM3367	4	24.09

S.No.	Marker Name	Chromosome	Position
34	RM470	4	28.09
35	RM3276	4	30.68
36	RM1153	4	32.84
37	RM3381	5	9.58
38	RM18371	5	14.23
39	Hv5-25	5	9.12
40	RM440	5	19.91
41	RM3476	5	23.91
42	Hv5-64	5	27.77
43	RM19291	6	1.21
44	RM204	6	3.16
45	RGNMS2220	6	30.98
46	RM528	6	26.17
47	RM2180	6	7.28
48	RM162	6	24.04
49	RM1261	6	17.57
50	RM7	7	9.83
51	RM5436	7	9.08
52	RM2	7	16.02
53	RM432	7	18.03
54	RM1132	7	23.98
55	RM47	7	25.81
56	RM22825	8	11.75
57	RM404	8	15.43
58	RM22807	8	10.79
59	RM25	8	4.38
60	RM22832	8	11.61
61	RM5637	8	21.23
62	RM5688	9	1.71
63	RM444	9	5.92
64	RM7364	9	9.56
65	Hv9-25	9	14.74
66	RM257	9	17.71
67	RM278	9	23.01
68	RM6100	10	10.89
69	RM474	10	1.81
70	RM467	10	13.48
71	RGNMS3297	10	16.25
72	RM286	11	0.38
73	RM1812	11	2.41
74	RM7226	11	14.51
75	RM26918	11	20.13
76	RM1227	12	27.38
77	RM247	12	3.19
78	RM519	12	19.9

Supplementary Table S2. Comprehensive list of annotated genes identified within the *qLPA8.1* QTL region.

S. No.	Locus ID	Position		Functions
		Start	End	
1	Os08g0175800	4419215	4419262	Non-protein coding transcript
2	Os08g0178000	4566329	4566665	Non-protein coding transcript
3	Os08g0178400	4592000	4592583	Non-protein coding transcript
4	Os08g0178650	4597056	4597381	Protein unknown function; wound-induced domain containing protein
5	Os08g0180950	4718736	4718969	Hypothetical conserved gene
6	Os08g0181925	4779112	4779498	Conserved hypothetical protein
7	Os08g0184700	4968125	4968451	Conserved hypothetical protein
8	Os08g0187600	5114107	5114622	Non-protein coding transcript
9	Os08g0188151	5165230	5165766	Hypothetical gene
10	Os08g0192001	5385370	5385976	Non-protein coding transcript
11	Os08g0192300	5400009	5400263	Conserved hypothetical protein
12	Os08g0199200	5735464	5735969	Conserved hypothetical protein
13	Os08g0201600	5893368	5893829	Non-protein coding transcript
14	Os08g0202500	5970251	5970875	Conserved hypothetical protein
15	Os08g0203201	5988432	5988931	Similar to SHR5-receptor-like kinase (Fragment)
16	Os08g0203325	6004271	6004772	Non-protein coding transcript
17	Os08g0203501	6022081	6022579	Non-protein coding transcript
18	Os08g0203750	6047428	6047963	Non-protein coding transcript
19	Os08g0208300	6319060	6319437	Conserved hypothetical protein
20	Os08g0211025	6527009	6527269	Hypothetical gene
21	Os08g0215900	6792260	6792917	Hypothetical gene
22	Os08g0216450	6852674	6852927	Conserved hypothetical protein
23	Os08g0223799	7495525	7495588	Non-protein coding transcript
24	Os08g0226666	7734190	7734875	Non-protein coding transcript
25	Os08g0227500	7778740	7778955	Similar to speckle-type POZ protein
26	Os08g0233000	8070577	8070804	Non-protein coding transcript
27	Os08g0235183	8207064	8207447	Similar to Thaumatin-like protein
28	Os08g0235550	8224155	8224982	Conserved hypothetical protein
29	Os08g0246700	8966304	8966798	Hypothetical gene
30	Os08g0249200	9111086	9111844	Similar to GDU1
31	Os08g0249600	9134106	9134700	Diacylglycerol kinase 6
32	Os08g0250300	9181114	9181517	Similar to predicted protein
33	Os08g0251000	9232201	9232818	Non-protein coding transcript
34	Os08g0252050	9327023	9327309	Non-protein coding transcript
35	Os08g0256100	9510326	9510824	Similar to OSIGBa0157K09-H0214G12.13

S. No.	Locus ID	Position		Functions
		Start	End	
36	Os08g0258600	9646326	9646835	Hypothetical gene
37	Os08g0260200	9773361	9773595	Non-protein coding transcript
38	Os08g0269500	10287058	10287839	Conserved hypothetical protein
39	Os08g0271600	10425433	10425813	Conserved hypothetical protein
40	Os08g0273783	10538061	10538823	Similar to Zinc finger, C2H2 type family protein, expressed
41	Os08g0274150	10567310	10567726	Hypothetical gene
42	Os08g0274775	10569519	10569899	Similar to phosphatidylinositol 3-and 4-kinase family protein
43	Os08g0280150	10906307	10906908	Conserved hypothetical protein
44	Os08g0280501	10962154	10962711	Non-protein coding transcript
45	Os08g0282700	11142234	11142856	Hypothetical gene
46	Os08g0289266	11510586	11510921	Similar to Histone H2A
47	Os08g0175200	4385769	4388881	Protein unknown function
48	Os08g0175450	4400972	4401019	Non-protein coding transcript
49	Os08g0175600	4411430	4417245	Similar to PolI-like DNA polymerase
50	Os08g0175700	4413202	4414401	Hypothetical protein
51	Os08g0176100	4430918	4434203	DNA/pantothenate metabolism flavoprotein C -terminal domain containing protein
52	Os08g0176200	4445655	4447532	Serine/threonine protein kinase-related domain containing protein
53	Os08g0176900	4505745	4508226	Similar to Transcription factor HBP-1b(C38) (Fragment)
54	Os08g0177000	4505997	4507323	Hypothetical protein
55	Os08g0177500	4546935	4549380	Conserved hypothetical protein
56	Os08g0177550	4550913	4557184	Conserved hypothetical protein
57	Os08g0177600	4552111	4556216	Similar to double-strand break repair protein MRE11
58	Os08g0177700	4562440	4565329	Similar to LSM7-like
59	Os08g0177800	4566815	4569933	Similar to RNase H domain-containing protein
60	Os08g0178200	4581432	4583489	Similar to Monosaccharide transporter 3
61	Os08g0178500	4593671	4594173	Similar to H0315F07.12 protein
62	Os08g0178600	4595778	4597522	Hypothetical gene
63	Os08g0178800	4610545	4612918	Hypothetical conserved gene
64	Os08g0179000	4630538	4638873	Protein kinase, catalytic domain domain containing protein
65	Os08g0179100	4635580	4638628	Hypothetical protein
66	Os08g0179150	4635505	4636456	Hypothetical conserved gene
67	Os08g0179400	4645325	4648753	Hypothetical conserved gene
68	Os08g0179900	4672775	4675317	Uncharacterised conserved protein UCP012943 domain containing protein
69	Os08g0180100	4692655	4696632	Similar to SET domain-containing protein.
70	Os08g0180300	4700668	4708354	DEAD-like helicase, N-terminal domain containing protein
71	Os08g0180500	4713705	4717694	Zinc finger, BED-type predicted domain containing protein

S. No.	Locus ID	Position		Functions
		Start	End	
72	Os08g0181750	4774914	4777902	Conserved hypothetical protein
73	Os08g0184600	4966415	4969131	Conserved hypothetical protein
74	Os08g0184800	4971046	4973826	Conserved hypothetical protein
75	Os08g0185701	5027574	5029922	Non-protein coding transcript
76	Os08g0185900	5034835	5035725	Conserved hypothetical protein
77	Os08g0186200	5046733	5048608	Hypothetical conserved gene
78	Os08g0187900	5145659	5146534	Ubiquitin-conjugating enzyme, E2 domain containing protein.
79	Os08g0188900	5186875	5187698	Germin-like protein 8-1, Disease resistance
80	Os08g0189100	5208410	5209440	Germin-like protein 8-3, Disease resistance
81	Os08g0189200	5222230	5223304	Germin-like protein 8-3, Disease resistance
82	Os08g0189300	5228827	5229949	Germin-like protein 8-4, Disease resistance
83	Os08g0189400	5233864	5234719	Germin-like protein 8-5, Disease resistance
84	Os08g0189600	5242497	5243656	Germin-like protein 8-7, Disease resistance
85	Os08g0189700	5248666	5249832	Germin-like protein 8-8, Disease resistance
86	Os08g0189850	5254364	5255230	Germin-like protein 8-9, Disease resistance
87	Os08g0189900	5260239	5261296	Germin-like protein 8-10, Disease resistance
88	Os08g0190100	5264245	5265340	Germin-like protein 8-11, Disease resistance
89	Os08g0190250	5274603	5277357	Hypothetical gene
90	Os08g0190300	5274970	5279294	Disease resistance protein domain containing protein
91	Os08g0190500	5314677	5315177	Conserved hypothetical protein
92	Os08g0190700	5315463	5317814	Conserved hypothetical protein
93	Os08g0191000	5322769	5325114	Auxin efflux carrier domain containing protein
94	Os08g0191100	5327664	5328165	Similar to Iron-responsive element binding protein (Fragment)
95	Os08g0191150	5328907	5329776	Non-protein coding transcript
96	Os08g0191200	5341449	5345391	VIRESCENT-ALBINO LEAF 1, glycinamide ribonucleotide synthetase
97	Os08g0191250	5344641	5345170	Hypothetical gene
98	Os08g0191300	5345885	5346658	Conserved hypothetical protein
99	Os08g0191466	5361078	5362944	Hypothetical gene
100	Os08g0191600	5364394	5366465	Core autophagy gene, Nitrogen use efficiency and yield
101	Os08g0191650	5371810	5375320	Hypothetical gene
102	Os08g0191700	5373362	5377125	GLYOXALASE I-11, glyoxalase I-11
103	Os08g0191800	5380261	5383885	Similar to Tyrosyl-tRNA synthetase
104	Os08g0191900	5384447	5388799	Pentatricopeptide repeat domain containing protein
105	Os08g0192100	5391860	5392486	Hypothetical conserved gene
106	Os08g0192200	5395517	5397475	Conserved hypothetical protein
107	Os08g0192400	5401162	5404366	Similar to DRP1 protein

S. No.	Locus ID	Position		Functions
		Start	End	
108	Os08g0192900	5422274	5426674	Nucleotide-binding, alpha-beta plait domain containing protein
109	Os08g0192950	5422644	5425791	Hypothetical protein
110	Os08g0193000	5432554	5434185	Conserved hypothetical protein
111	Os08g0193100	5435576	5439963	Polyprenyl synthetase-related domain containing protein
112	Os08g0193200	5441457	5442964	Hypothetical conserved gene
113	Os08g0193300	5446749	5448654	Hypothetical conserved gene
114	Os08g0193500	5452058	5453811	Hypothetical conserved gene
115	Os08g0193700	5466374	5469315	Similar to NBS-LRR disease resistance protein homologue (Fragment)
116	Os08g0193900	5474996	5477814	Cyclin-like F-box domain containing protein
117	Os08g0194350	5517737	5519259	F-box domain, Skp2-like domain containing protein
118	Os08g0194501	5532402	5536072	Conserved hypothetical protein
119	Os08g0194850	5543795	5545447	Hypothetical protein
120	Os08g0194900	5543765	5545891	Hypothetical conserved gene
121	Os08g0195000	5551365	5553193	F-box domain, Skp2-like domain containing protein
122	Os08g0195400	5563255	5564422	Protein of unknown function DUF3778 domain containing protein
123	Os08g0195800	5572031	5574384	Cyclin-like F-box domain containing protein
124	Os08g0195900	5576467	5578655	F-box domain, cyclin-like domain containing protein
125	Os08g0196200	5584231	5587025	Hypothetical conserved gene
126	Os08g0196900	5602622	5607315	Hypothetical conserved gene
127	Os08g0197025	5613322	5616590	Hypothetical gene
128	Os08g0197050	5613578	5616235	Hypothetical conserved gene
129	Os08g0197200	5623663	5626623	Conserved hypothetical protein
130	Os08g0197400	5633346	5636220	F-box domain, cyclin-like domain containing protein
131	Os08g0197700	5645714	5648057	Similar to Luminal binding protein 5 precursor (BiP 5)
132	Os08g0197800	5652681	5655649	Conserved hypothetical protein
133	Os08g0197900	5658727	5659464	Conserved hypothetical protein
134	Os08g0198000	5663578	5664043	HAT dimerisation domain containing protein
135	Os08g0198100	5672471	5676449	Zinc finger, BED-type predicted domain containing protein
136	Os08g0198200	5677979	5679084	Hypothetical conserved gene
137	Os08g0198300	5681460	5683088	Similar to speckle-type POZ protein
138	Os08g0198700	5691120	5697423	Similar to Glycolate oxidase (EC 1.1.3.15) (Fragment)
139	Os08g0198800	5706417	5709046	Conserved hypothetical protein
140	Os08g0198900	5713945	5719160	Conserved hypothetical protein
141	Os08g0199000	5720470	5723936	F-box domain, cyclin-like domain containing protein
142	Os08g0199100	5728025	5729681	F-box domain, cyclin-like domain containing protein
143	Os08g0199400	5751863	5753741	Similar to Stearoyl-acyl carrier protein desaturse

S. No.	Locus ID	Position		Functions
		Start	End	
144	Os08g0199525	5752141	5753244	ADP-glucose pyrophosphorylase, conserved site domain containing protein
145	Os08g0199775	5767787	5768582	Hypothetical gene
146	Os08g0200100	5787597	5789135	Ribonucleotide reductase-related domain containing protein
147	Os08g0200300	5806463	5808326	Similar to Photosystem II 10 kDa polypeptide (Fragment)
148	Os08g0200400	5811115	5816028	K Homology domain containing protein
149	Os08g0200500	5840300	5843516	Serine/threonine protein kinase domain containing protein
150	Os08g0201100	5864931	5865851	Conserved hypothetical protein
151	Os08g0201500	5878083	5879812	Similar to SHR5-receptor-like kinase (Fragment)
152	Os08g0201700	5893697	5894289	Protein kinase-like domain domain containing protein
153	Os08g0202200	5938279	5939791	Conserved hypothetical protein
154	Os08g0202350	5958903	5959364	Non-protein coding transcript
155	Os08g0202400	5964506	5968143	Disease resistance protein domain containing protein
156	Os08g0203100	5982519	5985990	Hypothetical conserved gene
157	Os08g0203150	5987781	5988339	Hypothetical gene
158	Os08g0203350	6006493	6007052	Conserved hypothetical protein
159	Os08g0203600	6032208	6039069	Hypothetical conserved gene
160	Os08g0203650	6037884	6038369	Non-protein coding transcript
161	Os08g0203700	6040528	6048561	Protein kinase, core domain containing protein
162	Os08g0203800	6055827	6058993	Cyclin-like F-box domain containing protein
163	Os08g0203900	6062772	6065354	UAA transporter family protein
164	Os08g0204632	6102975	6105362	Conserved hypothetical protein
165	Os08g0204800	6112375	6115612	Conserved hypothetical protein
166	Os08g0205000	6121845	6123652	Transferase domain containing protein
167	Os08g0205200	6142162	6143084	Delayed-early response protein/equilibrative nucleoside transporter family protein
168	Os08g0205300	6155162	6157777	Hypothetical conserved gene
169	Os08g0205500	6161997	6163054	Heavy metal-associated domain, HMA domain containing protein
170	Os08g0205650	6162026	6163058	Hypothetical protein
171	Os08g0205800	6168352	6169288	Hypothetical conserved gene
172	Os08g0205900	6170105	6175794	Similar to Viroid RNA-binding protein (Fragment)
173	Os08g0206466	6210108	6211145	Hypothetical gene
174	Os08g0206500	6212073	6217326	Similar to HAP5 subunit of HAP complex
175	Os08g0206800	6233507	6234131	Similar to cDNA clone:001-034-C01, full insert sequence
176	Os08g0206950	6242130	6243735	Similar to Pectinesterase
177	Os08g0207000	6244128	6245111	Similar to 40S ribosomal protein S11
178	Os08g0207300	6251353	6252206	Conserved hypothetical protein
179	Os08g0207800	6281221	6286663	Peptidase aspartic, catalytic domain containing protein

S. No.	Locus ID	Position		Functions
		Start	End	
180	Os08g0207951	6303848	6304320	Hypothetical protein
181	Os08g0208200	6313873	6314560	Peptidase aspartic, catalytic domain containing protein
182	Os08g0208700	6334289	6339617	Zinc finger, BED-type predicted domain containing protein
183	Os08g0209000	6358448	6359451	Conserved hypothetical protein
184	Os08g0210300	6432276	6434158	Similar to H0502G05.7 protein
185	Os08g0213200	6585028	6586115	Hypothetical conserved gene
186	Os08g0213816	6616927	6617180	Conserved hypothetical protein
187	Os08g0214233	6626915	6627830	Conserved hypothetical protein
188	Os08g0214300	6670593	6671933	Hypothetical conserved gene
189	Os08g0214600	6712297	6713348	Conserved hypothetical protein
190	Os08g0215300	6734412	6739686	Conserved hypothetical protein
191	Os08g0216000	6745946	6747840	Hypothetical conserved gene
192	Os08g0216050	6827896	6829236	Hypothetical conserved gene
193	Os08g0216300	6842089	6843002	Conserved hypothetical protein
194	Os08g0216900	6891099	6893671	Conserved hypothetical protein
195	Os08g0217050	6942146	6948943	Similar to H0124E07.9 protein
196	Os08g0217800	7085914	7086571	Hypothetical protein
197	Os08g0218700	7165471	7166548	Conserved hypothetical protein
198	Os08g0219100	7203560	7205848	Similar to cDNA clone:J023038L08, full insert sequence
199	Os08g0220400	7325157	7327754	Pectin lyase fold/virulence factor domain containing protein.
200	Os08g0220500	7325507	7326193	Hypothetical protein
201	Os08g0221300	7363845	7367943	Conserved hypothetical protein
202	Os08g0222100	7410180	7411388	Conserved hypothetical protein
203	Os08g0222800	7451363	7452016	Similar to cDNA clone:J023038L08, full insert sequence
204	Os08g0223900	7540127	7547972	Bicyclic triterpene poaceatapetol synthase, Pollen coat formation
205	Os08g0224100	7553773	7558719	Similar to Serine/thronine protein kinase-like protein
206	Os08g0224150	7554714	7558408	Hypothetical protein
207	Os08g0224500	7583487	7588332	Similar to 3-glucanase
208	Os08g0224700	7591562	7596941	Similar to 26S proteasome subunit RPN2a
209	Os08g0224901	7607234	7609254	Non-protein coding transcript
210	Os08g0225000	7612317	7614842	β-hydroxyacyl-ACP dehydratase, Regulation of fatty acid synthesis, Chloroplast development
211	Os08g0225700	7686865	7687583	Hypothetical conserved gene
212	Os08g0225900	7691858	7693642	Hypothetical conserved gene
213	Os08g0226000	7693777	7694865	Similar to speckle-type POZ protein
214	Os08g0226100	7698342	7699299	Hypothetical conserved gene
215	Os08g0226166	7710739	7711320	Similar to Cytochrome P450 CYP76H18
216	Os08g0226232	7711067	7716513	Hypothetical protein

S. No.	Locus ID	Position		Functions
		Start	End	
217	Os08g0226300	7714178	7716625	Similar to Cytochrome P450 family protein
218	Os08g0226400	7717968	7719211	Hypothetical conserved gene
219	Os08g0226600	7730270	7733632	Hypothetical gene
220	Os08g0226800	7740247	7741464	Similar to speckle-type POZ protein
221	Os08g0227100	7756668	7758169	Kelch related domain containing protein
222	Os08g0227200	7760699	7762222	Kelch related domain containing protein
223	Os08g0227400	7777850	7778536	Similar to speckle-type POZ protein
224	Os08g0227900	7803012	7807496	Hypothetical conserved gene
225	Os08g0228100	7820316	7821765	Non-protein coding transcript
226	Os08g0228200	7834796	7836523	BTB/POZ-like domain containing protein
227	Os08g0229200	7882502	7883098	Non-protein coding transcript
228	Os08g0229500	7892281	7893913	Hypothetical protein
229	Os08g0229601	7892344	7894459	Hypothetical conserved gene
230	Os08g0230000	7911799	7916363	Conserved hypothetical protein
231	Os08g0230500	7964016	7968546	Protein of unknown function DUF537 family protein
232	Os08g0230600	7972782	7973478	LIM, zinc-binding domain containing protein
233	Os08g0230800	7982512	7985309	Hypothetical conserved gene
234	Os08g0230900	7988597	7989786	Similar to Subtilisin protease (Fragment)
235	Os08g0231100	7991097	7993729	Hypothetical protein
236	Os08g0231400	7994404	7995717	Germin-like protein 8-12, Disease resistance
237	Os08g0232000	8011977	8014326	Similar to Glucose-6-phosphate isomerase
238	Os08g0232700	8061317	8063491	Exo70 exocyst complex subunit family protein
239	Os08g0233300	8104943	8107543	Similar to cDNA clone:J013061K19, full insert sequence
240	Os08g0233900	8147380	8148509	Conserved hypothetical protein
241	Os08g0234000	8153389	8155815	Similar to 60S ribosomal protein L7-2
242	Os08g0234050	8154564	8155536	Conserved hypothetical protein
243	Os08g0234100	8156590	8158428	Conserved hypothetical protein
244	Os08g0234400	8165310	8168702	Hypothetical gene
245	Os08g0234700	8175612	8177396	Conserved hypothetical protein
246	Os08g0235400	8216495	8219321	Conserved hypothetical protein
247	Os08g0235600	8230111	8235214	Hypothetical conserved gene
248	Os08g0235651	8235249	8236739	NB-ARC domain containing protein
249	Os08g0235800	8258523	8259611	Similar to WRKY transcription factor 25
250	Os08g0236400	8283843	8285334	Epidermal growth factor-like, type 3 domain containing protein
251	Os08g0236700	8298118	8299493	Similar to Exonuclease
252	Os08g0236800	8300327	8302290	Conserved hypothetical protein
253	Os08g0236866	8303870	8307351	Hypothetical conserved gene

S. No.	Locus ID	Position		Functions
		Start	End	
254	Os08g0237000	8328017	8329241	Xyloglucan endotransglycosylase/hydrolase protein 8 precursor
255	Os08g0237100	8335699	8339610	Conserved hypothetical protein
256	Os08g0237200	8342253	8346555	Mannose-1-phosphate guanyltransferase
257	Os08g0237500	8342408	8344154	Non-protein coding transcript
258	Os08g0237800	8384563	8385805	Similar to Xyloglucan endotransglycosylase (Fragment)
259	Os08g0238100	8390973	8392828	Similar to Pentatricopeptide repeat protein PPR986-12
260	Os08g0238200	8394276	8397823	Dienelactone hydrolase domain containing protein
261	Os08g0238700	8415405	8417869	Conserved hypothetical protein
262	Os08g0239000	8424284	8425290	Complex 1 LYR protein family protein
263	Os08g0239900	8464913	8469555	Similar to Flavonol 4-sulfotransferase
264	Os08g0240000	8472521	8475589	Similar to STF-1 (Fragment)
265	Os08g0240500	8488370	8489782	Glycoside hydrolase, family 16 domain containing protein
266	Os08g0240566	8494099	8495316	Similar to OSIGBa0118P15.7 protein
267	Os08g0240966	8527768	8528653	Non-protein coding transcript
268	Os08g0241300	8570830	8575238	Similar to OSIGBa0118P15.3 protein
269	Os08g0241400	8581992	8584058	Similar to protein binding protein
270	Os08g0241800	8613746	8615699	Similar to Plasma membrane H+-ATPase
271	Os08g0241900	8615720	8617846	Hypothetical protein
272	Os08g0242900	8681628	8686438	Afadin/alpha-actinin-binding domain containing protein
273	Os08g0243001	8681711	8683505	Non-protein coding transcript
274	Os08g0243100	8703799	8708888	4'-phosphopantetheinyl transferase domain containing protein
275	Os08g0243500	8760402	8765182	Similar to NADPH-cytochrome P450 oxydoreductase isoform 2
276	Os08g0243900	8795683	8797831	Mu2 adaptin subunit (AP50) of AP2 domain containing protein
277	Os08g0244100	8802820	8806880	Syntaxin 6, N-terminal domain containing protein
278	Os08g0244400	8811775	8816699	SET domain containing protein
279	Os08g0244500	8837400	8839354	Similar to hydrolase, hydrolyzing O-glycosyl compounds
280	Os08g0244625	8838319	8839100	Non-protein coding transcript
281	Os08g0244750	8850008	8852859	Conserved hypothetical protein
282	Os08g0244800	8856335	8858424	Non-protein coding transcript
283	Os08g0245200	8874179	8878862	4-coumarate:coenzyme A ligase, Lignin biosynthesis
284	Os08g0245301	8874383	8878050	Hypothetical gene
285	Os08g0246001	8915335	8917400	Hypothetical gene
286	Os08g0246100	8922657	8926442	Hypothetical conserved gene
287	Os08g0246400	8955294	8959576	Similar to Ubiquinol-cytochrome c reductase complex 14 kDa protein
288	Os08g0246500	8963793	8964481	Similar to Mitochondrial GTPase

S. No.	Locus ID	Position		Functions
		Start	End	
289	Os08g0246550	8964090	8965199	Hypothetical gene
290	Os08g0246950	8977536	8978715	Hypothetical protein
291	Os08g0247600	9001801	9005304	Leucine-rich repeat, N-terminal domain containing protein
292	Os08g0248000	9030748	9031356	Similar to predicted protein
293	Os08g0248400	9050060	9051612	Pentatricopeptide repeat domain containing protein
294	Os08g0248800	9071719	9075883	Similar to Aspartate carbamoyltransferase 3, chloroplast precursor
295	Os08g0248900	9081566	9086469	Similar to ADP-ribosylation factor 3
296	Os08g0249000	9098485	9099878	Zinc finger, B-box domain containing protein
297	Os08g0249100	9101238	9105746	Serine/threonine protein kinase-related domain containing protein
298	Os08g0249300	9123551	9126818	Hypothetical protein
299	Os08g0249400	9126454	9127450	Hypothetical conserved gene
300	Os08g0249675	9134388	9135396	Hypothetical protein
301	Os08g0249900	9165968	9167452	Similar to Gibberellin 20 oxidase 2
302	Os08g0250100	9174654	9178792	Hypothetical conserved gene
303	Os08g0250900	9224375	9228110	Homologue of the Arabidopsis thaliana Suppressor of MAX2-1
304	Os08g0253800	9384129	9387669	Similar to Cellulose synthase-like family C3 protein (Fragment)
305	Os08g0254200	9395073	9397980	Conserved hypothetical protein
306	Os08g0254600	9411335	9416401	Similar to cyclic nucleotide-gated ion channel 14
307	Os08g0254900	9424760	9428473	Similar to Glutamyl-tRNA reductase binding protein
308	Os08g0255140	9440105	9441435	Hypothetical gene
309	Os08g0256000	9507285	9508548	Conserved hypothetical protein
310	Os08g0256700	9538125	9539756	Conserved hypothetical protein
311	Os08g0256750	9545388	9548984	Hypothetical gene
312	Os08g0259100	9692697	9697289	Conserved hypothetical protein
313	Os08g0260600	9784270	9786922	Tetraspanin domain containing protein
314	Os08g0260800	9795807	9798597	Similar to NB-ARC domain containing protein
315	Os08g0261000	9824627	9828471	NB-ARC domain containing protein
316	Os08g0261100	9831006	9834047	Protein of unknown function DUF1218 family protein
317	Os08g0262500	9921522	9923218	Cytochrome P450 family protein
318	Os08g0263100	9964987	9965740	Conserved hypothetical protein
319	Os08g0263300	9976976	9979975	Similar to cDNA clone:J013108L16, full insert sequence
320	Os08g0263400	9984345	9985265	Hypothetical protein
321	Os08g0264700	10051525	10053112	Hypothetical conserved gene
322	Os08g0265000	10062034	10064824	Hypothetical gene
323	Os08g0266200	10131802	10135064	Similar to T24D18.25 protein
324	Os08g0266225	10136783	10137582	Conserved hypothetical protein

S. No.	Locus ID	Position		Functions
		Start	End	
325	Os08g0266400	10142326	10144699	Leucine-rich repeat, N-terminal domain containing protein
326	Os08g0266600	10152106	10156715	WD40/YVTN repeat-like domain containing protein
327	Os08g0266700	10160233	10165699	Rad21/Rec8 like protein, C-terminal domain containing protein
328	Os08g0267300	10190139	10193202	Peptidase A1 domain containing protein
329	Os08g0267425	10190145	10191481	Non-protein coding transcript
330	Os08g0268000	10221804	10222906	Conserved hypothetical protein
331	Os08g0268550	10257621	10259312	Conserved hypothetical protein
332	Os08g0268900	10265432	10268892	Hypothetical conserved gene
333	Os08g0269300	10284185	10285114	Similar to H0311C03.5 protein
334	Os08g0269600	10291954	10292821	EGF-like region domain containing protein
335	Os08g0269700	10294239	10295050	Conserved hypothetical protein
336	Os08g0270400	10355413	10358378	Similar to Cinnamyl alcohol dehydrogenase
337	Os08g0270500	10360387	10364290	Conserved hypothetical protein
338	Os08g0270800	10367168	10369143	Conserved hypothetical protein
339	Os08g0270900	10372707	10373655	Conserved hypothetical protein
340	Os08g0271400	10410093	10411679	Hypothetical protein
341	Os08g0272000	10444349	10446816	Similar to GAG1At protein
342	Os08g0272200	10453730	10456174	Pentatricopeptide repeat domain containing protein
343	Os08g0272800	10473629	10474373	Hypothetical gene
344	Os08g0273000	10491641	10494280	Conserved hypothetical protein
345	Os08g0273600	10534399	10539928	Non-protein coding transcript
346	Os08g0275200	10590895	10592947	Protein kinase, core domain containing protein
347	Os08g0275600	10591039	10592636	Hypothetical protein
348	Os08g0276000	10621769	10625430	Similar to Transmembrane 9 superfamily protein member 4
349	Os08g0276100	10630309	10633641	Similar to NDF1 (NDH-DEPENDENT CYCLIC ELECTRON FLOW 1)
350	Os08g0276200	10637548	10639603	Similar to WRKY transcription factor 44
351	Os08g0276400	10655519	10658544	Serine/threonine protein kinase domain containing protein
352	Os08g0277300	10723268	10726789	Similar to Flavonol 4-sulfotransferase
353	Os08g0278501	10781685	10782141	Hypothetical protein
354	Os08g0278750	10804531	10805024	Similar to nucleotidyltransferase family protein
355	Os08g0278900	10810395	10813333	MIR domain containing protein
356	Os08g0280125	10900154	10903100	Hypothetical protein
357	Os08g0280600	10962195	10967484	Reticulon domain containing protein
358	Os08g0281500	11030245	11031588	DNA-binding pseudobarrel domain domain containing protein
359	Os08g0281600	11035931	11037288	F-box domain, cyclin-like domain containing protein
360	Os08g0282100	11064461	11066098	DNA-binding pseudobarrel domain domain containing protein

S. No.	Locus ID	Position		Functions
		Start	End	
361	Os08g0282200	11074545	11078779	Hypothetical protein
362	Os08g0282400	11102726	11108224	Similar to Alpha-SNAP (Fragment)
363	Os08g0283000	11158562	11159207	Similar to H0315A08.1 protein
364	Os08g0283300	11170433	11171225	Similar to H0315A08.1 protein
365	Os08g0283600	11178543	11179368	Similar to H0315A08.1 protein
366	Os08g0283900	11201775	11202600	Similar to H0315A08.1 protein
367	Os08g0284500	11245647	11246472	Similar to H0315A08.1 protein
368	Os08g0285200	11263369	11266927	WD40 repeat-like domain containing protein
369	Os08g0285301	11267321	11268866	Conserved hypothetical protein
370	Os08g0285350	11267744	11268082	Hypothetical conserved gene
371	Os08g0285600	11274674	11279209	Similar to phytosulfokine receptor
372	Os08g0286100	11310539	11311944	Similar to predicted protein
373	Os08g0286500	11321679	11323563	Hypothetical conserved gene
374	Os08g0287200	11374384	11375591	Hypothetical conserved gene
375	Os08g0288000	11404746	11409808	Conserved hypothetical protein
376	Os08g0288400	11460357	11462712	Similar to transmembrane 9 superfamily protein member 2
377	Os08g0288500	11463136	11468530	Similar to G-patch domain containing protein
378	Os08g0290200	11564027	11565163	Protein of unknown function DUF313 domain containing protein
379	Os08g0290400	11583484	11587229	Conserved hypothetical protein
380	Os08g0290700	11607393	11608814	Winged helix repressor DNA-binding domain containing protein
381	Os08g0176633	4468741	4472698	Conserved hypothetical protein
382	Os08g0177300	4523794	4538717	HSA domain containing protein
383	Os08g0178100	4571022	4580739	Pep3/Vps18/deep orange domain containing protein
384	Os08g0178300	4586478	4591217	Protein of unknown function DUF2050
385	Os08g0178900	4617579	4620400	Homeodomain-like domain containing protein
386	Os08g0180000	4676292	4680426	mRNA capping enzyme, large subunit family protein
387	Os08g0180400	4709755	4710209	Similar to Kinesin-like protein
388	Os08g0182400	4817967	4820693	Conserved hypothetical protein
389	Os08g0184300	4947263	4952151	Hypothetical protein
390	Os08g0187500	5109268	5116778	Similar to Roc1
391	Os08g0187700	5119189	5128123	ENTH/VHS domain containing protein
392	Os08g0187800	5139674	5143593	Glucose 6-phosphate/phosphate translocator, Starch biosynthesis
393	Os08g0188000	5147424	5150332	RNA-binding, CRM domain domain containing protein
394	Os08g0189500	5238999	5240148	Germin-like protein 8-6, Disease resistance
395	Os08g0190200	5268917	5273165	Similar to cDNA clone:J033051E20, full insert sequence
396	Os08g0190225	5269409	5272023	Hypothetical protein

S. No.	Locus ID	Position		Functions
		Start	End	
397	Os08g0190800	5318646	5320835	Postsynaptic protein CRIPT
398	Os08g0190901	5318774	5320582	Non-protein coding transcript
399	Os08g0191400	5349923	5350343	Hypothetical gene
400	Os08g0191433	5352105	5363276	Starch synthase, Starch biosynthesis
401	Os08g0192800	5418272	5420390	Bromodomain containing protein
402	Os08g0193600	5457347	5460486	Hypothetical conserved gene
403	Os08g0194000	5479726	5481493	F-box domain, Skp2-like domain containing protein
404	Os08g0195650	5569446	5570315	Conserved hypothetical protein
405	Os08g0197000	5609250	5612337	Cyclin-like F-box domain containing protein
406	Os08g0197100	5618541	5621553	Cyclin-like F-box domain containing protein
407	Os08g0197300	5628896	5632250	F-box domain, cyclin-like domain containing protein
408	Os08g0197500	5638269	5643201	Cyclin-like F-box domain containing protein
409	Os08g0197950	5659559	5661224	Hypothetical conserved gene
410	Os08g0199300	5746738	5751829	Similar to YyaF/YCHF TRANSFAC/OBG family small GTpase plus RNA binding domain TGS (Fragment)
411	Os08g0200600	5846866	5850647	NAC transcription factor, Negative regulation of drought tolerance
412	Os08g0200750	5846926	5850631	Hypothetical protein
413	Os08g0202300	5942660	5960134	Hypothetical conserved gene
414	Os08g0203400	6015745	6023225	Protein kinase, core domain containing protein
415	Os08g0205100	6127418	6131390	Disease resistance protein domain containing protein
416	Os08g0205125	6127823	6131014	Hypothetical protein
417	Os08g0205150	6137622	6141504	CC-NBS-LRR protein, Cold stress signaling and response
418	Os08g0205175	6138088	6141373	Hypothetical protein
419	Os08g0205400	6159123	6161383	Cu chaperone, Root-to-shoot Cu translocation, Distribution of Cu from old leaves to developing tissues and seeds
420	Os08g0206400	6206039	6211417	Similar to Potassium transporter 18
421	Os08g0206600	6218688	6224037	Similar to AICARFT/IMPCHase bienzyme family protein
422	Os08g0206650	6218749	6223799	Hypothetical protein
423	Os08g0206700	6226356	6231302	DNA-binding, integrase-type domain containing protein
424	Os08g0206900	6237449	6242078	UDP-N-acetylglucosamine pyrophosphorylase 1, Regulation of leaf senescence, Defence responses
425	Os08g0207401	6267772	6270409	Hypothetical gene
426	Os08g0207500	6267823	6270904	Similar to Zinc transporter ZIP1 (Fragment)
427	Os08g0207600	6272214	6280917	Similar to GTP-binding protein
428	Os08g0208400	6319161	6324373	Conserved hypothetical protein
429	Os08g0211750	6553984	6585906	Conserved hypothetical protein
430	Os08g0214900	6719347	6720055	Hypothetical conserved gene
431	Os08g0215200	6728264	6732694	Conserved hypothetical protein
432	Os08g0215400	6736981	6742879	Conserved hypothetical protein

S. No.	Locus ID	Position		Functions
		Start	End	
433	Os08g0216600	6866467	6870682	Similar to cDNA clone:J013022L16, full insert sequence
434	Os08g0220600	7339066	7348077	Fibronectin, type III domain containing protein
435	Os08g0223700	7488653	7492184	EMB1135 (EMBRYO DEFECTIVE 1135)
436	Os08g0223833	7501641	7510161	Similar to H0702G05.10 protein
437	Os08g0224000	7545583	7551989	NB-ARC domain containing protein
438	Os08g0224200	7559100	7563171	Similar to H0702G05.3 protein
439	Os08g0224250	7559814	7560619	Non-protein coding transcript
440	Os08g0224300	7568558	7573900	Determination of the grain yield, Modulation of nitrogen utilization
441	Os08g0224800	7605599	7610209	Hypothetical conserved gene
442	Os08g0225050	7616789	7620327	Hypothetical gene
443	Os08g0225100	7616789	7620106	Similar to predicted protein
444	Os08g0225400	7652630	7664411	Similar to OSIGBa0113I13.9 protein
445	Os08g0230100	7919850	7921536	Hypothetical conserved gene
446	Os08g0230200	7939705	7944306	Similar to cysteine-type endopeptidase/ ubiquitin thiolesterase
447	Os08g0230300	7947639	7951629	Hypothetical conserved gene
448	Os08g0230350	7948087	7951633	Non-protein coding transcript
449	Os08g0232100	8018574	8021328	Hypothetical gene
450	Os08g0233400	8107778	8116271	Ankyrin repeat domain containing protein
451	Os08g0233600	8118287	8120253	Hypothetical conserved gene
452	Os08g0234200	8159973	8162772	Conserved hypothetical protein
453	Os08g0235650	8229426	8237973	Hypothetical conserved gene
454	Os08g0236900	8307815	8311773	Conserved hypothetical protein
455	Os08g0238351	8405972	8410112	Non-protein coding transcript
456	Os08g0238500	8406018	8410016	Similar to Dienelactone hydrolase family
457	Os08g0238600	8412054	8420579	Similar to Dienelactone hydrolase family
458	Os08g0239300	8437323	8445916	Dienelactone hydrolase domain containing protein
459	Os08g0240200	8479583	8481489	Conserved hypothetical protein
460	Os08g0240800	8508463	8518590	Similar to Actin filament bundling protein P-115-ABP
461	Os08g0241600	8589245	8594472	Similar to apospory-associated protein C
462	Os08g0242400	8639193	8640760	Similar to WUSCHEL-related homeobox 8
463	Os08g0242700	8668958	8672734	Similar to uridylyltransferase-related
464	Os08g0242800	8677955	8681460	Similar to Sigma factor SIG6
465	Os08g0243600	8767789	8771574	Haloacid dehalogenase-like hydrolase domain containing protein
466	Os08g0243866	8789627	8792452	Similar to chromatin remodeling complex subunit
467	Os08g0245400	8879953	8888141	Pyridoxal phosphate-dependent transferase, major region, subdomain 1 domain containing protein
468	Os08g0246300	8939500	8953088	NB-ARC domain containing protein

S. No.	Locus ID	Position		Functions
		Start	End	
469	Os08g0246351	8939535	8940950	Hypothetical protein
470	Os08g0246800	8969014	8970904	Conserved hypothetical protein
471	Os08g0247700	9016956	9020228	Protein kinase, catalytic domain domain containing protein
472	Os08g0248100	9039735	9043242	Protein kinase, core domain containing protein
473	Os08g0248300	9049938	9051200	Hypothetical protein
474	Os08g0248700	9069082	9070671	Similar to Blind
475	Os08g0249501	9128282	9136609	Hypothetical protein
476	Os08g0249750	9138740	9145238	Non-protein coding transcript
477	Os08g0250200	9177347	9180467	ATPase, F1 complex, epsilon subunit, mitochondrial family protein
478	Os08g0253150	9339279	9343379	Conserved hypothetical protein
479	Os08g0254500	9405688	9414700	Similar to Preprotein translocase secY subunit, chloroplast precursor
480	Os08g0255020	9439937	9441162	Conserved hypothetical protein
481	Os08g0255500	9477453	9482623	Similar to BRASSINOSTEROID INSENSITIVE 1-associated receptor kinase 1
482	Os08g0256300	9517361	9522020	Similar to OSIGBa0135A16.2 protein
483	Os08g0258200	9628599	9632710	Ankyrin domain containing protein
484	Os08g0260000	9757448	9760662	Similar to N-acetyltransferase
485	Os08g0260400	9779953	9782015	Conserved hypothetical protein
486	Os08g0263000	9958741	9960475	Similar to Cytochrome P450-like protein (CYP86B1)
487	Os08g0263600	9985994	9990182	Conserved hypothetical protein
488	Os08g0265300	10071862	10080093	Disease resistance protein domain containing protein
489	Os08g0266300	10138571	10141527	Disease resistance protein domain containing protein
490	Os08g0267800	10217767	10220568	Cyclin-like F-box domain containing protein
491	Os08g0269000	10269576	10270739	Hypothetical conserved gene
492	Os08g0270000	10314550	10320983	F-box domain, cyclin-like domain containing protein
493	Os08g0270200	10329433	10335439	Exosome-associated family protein
494	Os08g0274700	10567331	10578007	Similar to TTN10
495	Os08g0277200	10718581	10720791	Similar to Cinnamoyl-CoA reductase
496	Os08g0277900	10759593	10763435	Similar to Syntaxin 52 (AtSYP52)
497	Os08g0278100	10767792	10771186	Osteocrin domain containing protein
498	Os08g0278966	10817993	10821522	Similar to predicted protein
499	Os08g0280200	10922470	10936171	Tensin phosphatase, C2 domain domain containing protein
500	Os08g0280300	10938444	10945133	Hypothetical conserved gene
501	Os08g0282000	11055447	11062439	Tetraspanin domain containing protein
502	Os08g0282500	11126808	11130613	WD40 repeat-like domain containing protein
503	Os08g0284200	11209874	11210515	Similar to H0315A08.1 protein
504	Os08g0285100	11258610	11260127	Conserved hypothetical protein

S. No.	Locus ID	Position		Functions
		Start	End	
505	Os08g0288050	11437989	11440989	Hypothetical conserved gene
506	Os08g0288200	11442670	11453068	Adenylate kinase family protein
507	Os08g0289000	11487287	11491371	Conserved hypothetical protein
508	Os08g0289400	11512090	11528238	SNF2-related domain containing protein
509	Os08g0290000	11548007	11554951	Nuclear-localized pentatricopeptide repeat (PPR) protein
510	Os08g0290100	11558486	11563711	Pentatricopeptide repeat containing protein
511	Os08g0290500	11587812	11590913	Similar to PHD finger family protein
512	Os08g0175300	4391050	4400757	PolI-like DNA polymerase
513	Os08g0176800	4496687	4502242	Similar to mRNA-associated protein mrnp 41 (Rae1 protein homolog)
514	Os08g0178700	4599751	4604714	Similar to Calmodulin-binding diacylglycerol kinase
515	Os08g0183900	4897613	4900341	Similar to dihydroflavonol-4-reductase
516	Os08g0196700	5597606	5601049	HAP2 subunit of HAP complex, Nuclear Factor Y (NF-YA) transcription factor, Drought stress tolerance
517	Os08g0213400	6598294	6603374	Conserved hypothetical protein
518	Os08g0223766	7495162	7500893	Similar to H0702G05.10 protein
519	Os08g0240600	8497291	8500100	Similar to predicted protein
520	Os08g0250700	9191621	9201077	Thioredoxin domain 2 containing protein
521	Os08g0254300	9399521	9402233	Similar to *OsIGBa0112M24.2* protein
522	Os08g0265500	10097893	10103955	Similar to ATPase
523	Os08g0267000	10198285	10201713	F-box domain, Skp2-like domain containing protein
524	Os08g0267450	10198947	10201019	Hypothetical gene
525	Os08g0269800	10296961	10300697	Similar to Ubiquitin-protein ligase
526	Os08g0278600	10796069	10803842	Similar to complex 1 protein containing protein
527	Os08g0280100	10899946	10909610	Similar to Phytase
528	Os08g0287800	11399711	11402862	Hypothetical conserved gene
529	Os08g0185300	4999380	5002096	Conserved hypothetical protein
530	Os08g0203300	5998167	6005500	Similar to SHR5-receptor-like kinase (Fragment)
